# Two new species of *Floresorchestia* (Crustacea, Amphipoda, Talitridae) in Thailand

**DOI:** 10.3897/zookeys.635.10454

**Published:** 2016-11-23

**Authors:** Koraon Wongkamhaeng, Pongrat Dumrongrojwattana, Manasawan Saengsakda Pattaratumrong

**Affiliations:** 1 Marine and Coastal Resources Institute, Prince of Songkla University, Thailand; 2 Department of Biology, Faculty of Science, Burapha University, Thailand

**Keywords:** Amphipoda, Crustacean, Floresorchestia, new species, Talitridae, Thailand

## Abstract

The beach-hopper and land-hopper genus *Floresorchestia* Bousfield, 1984 is most widespread in terrestrial and marine littoral habitats and has been recorded from the South African coasts through to tropical Indo-Pacific and Caribbean Sea. In Thailand, there is only *Floresorchestia samroiyodensis* Azman, Wongkamhaeng & Dumrongrojwattana, 2014 reported from the swamp of Prachuab Kiri Khan, southern Thailand. In this work, two new species of *Floresorchestia* from Phutsa Reservoir in Nakhon Ratchasima and the man-made swamp in Burapha University are described. The new species are characterised by the mandible left lacinia mobilis 4-dentate; the posterior margin of merus, carpus and propodus covered in palmate setae; the uropod 3 peduncle with two robust setae and the telson longer than broad. The characters of the specimens are described and illustrated in this paper. All specimens are deposited in the Princess Maha Chakri Sirindhorn Natural History Museum, Prince of Songkla University, Thailand.

## Introduction

The talitrid amphipod genus *Floresorchestia* Bousfield, 1984 is most widespread in littoral and terrestrial habitats. They have been recorded from the South African coast through to tropical Indo Pacific and Caribbean Sea. The amphipod can be recognised from the number of stridulating organs above the ventral margin (epimera 1–3, 2 or 2–3). Members of this genus occupy a variety of habitat such as beach (beach-hoppers) or land (land-hoppers) or stream (stream-hoppers) (Lowry and Springthorpe 2015). The genus *Floresorchestia* has established by Bousfield (1984) for a group of amphipod species with epimeral slit. After that, Serejo (2004) proposed the classification for superfamily Talitroidea and *Floresorchestia* is placed into the family Talitridae, the lineage of amphipods that invaded the terrestrial habitat. The *Floresorchestia* has recently been revised by Lowry and Springthorpe (2015). From that, four species are redescribed based on neotypes and lectotypes newly established from this work with the description of nine new species.

In Southeast Asia, thirteen species of *Floresorchestia* have been recorded. There are both supralittoral (seven species) and terrestrial groups (six species). The supralittoral species have been reported from the Gulf of Thailand (*Floresorchestia* sp. 3), (Bussarawich 1985); South China Sea (*Floresorchestia* sp. 1); Malaysian Peninsula (*Floresorchestia seringat* Lowry & Springthorpe, 2015); Indonesian Waters (*Floresorchestia floresiana* (Weber, 1892) and *Floresorchestia laurenae* Lowry & Springthorpe, 2015) and Taiwan Waters (*Floresorchestia anpingensis* Miyamoto & Morino , 2008 and *Floresorchestia oluanpi* Lowry & Springthorpe, 2015). The terrestrial species have also been recorded from different areas including *Floresorchestia samroiyodenesis* Azman, Wongkamhaeng & Dumrongrojwattana, 2014 from Thailand; *Floresorchestia malayensis* (Tattersall,1922) from Singapore and Malaysia; *Floresorchestia thienemanni* (Schellenberg, 1931) from Indonesia; *Floresorchestia vugiaensis* (Dang & Le, 2011) and *Floresorchestia hanoiensis* Hou & Li, 2003 in Vietnam and *Floresorchestia yehyuensis* Miyamoto & Morino, 2008 from Taiwan. In this study, two new 4-dentate cuspidactylate amphipods from terrestrial swamps and supralittoral have been discovered from north-eastern and eastern Thailand.

## Material and methods

This study is based upon material collected from leaf litter of Phutsa Reservoir in Nakhon Ratchasima, north-eastern Thailand and a man-made swamp in Burapha University, eastern Thailand in March and April 2016 respectively. Samples were collected using hand-nets and were then carefully transferred into plastic containers and fixed in 10% buffered formalin. In the laboratory, amphipod specimens were sorted out and stored in 70% alcohol. The animals were then examined under a compound microscope and later selected for dissection. The appendages of the dissected specimens were examined and figures were produced using camera lucida attached to an Olympus CH30 light microscope. The pencil drawings were scanned and digitally inked using a WACOM bamboo CTH-970 graphics board following the method described in Coleman (2003). Setal and mouthpart classifications were made following Watling (1989).

### Abbreviations for the figures

A
 antenna 


G
 gnathopod 


HD
 head 


LL
 lower lip 


MD
 mandible 


MX
 maxilla 


MP
 maxilliped 


P
 pereopod 


Pl
 pleopod 


T
 telson 


U
 uropod 


UR
 urosome 


UL
 upper lip 


R
 right 


L
 eft 


♂ male


♀ female


Type material has been deposited at Prince of Songkla University Zoological Collection (PSUZC).

## Systematic Results

### Suborder Senticauda Lowry & Myers, 2013a Infraorder Talitrida Rafinesque, 1815, Serejo 2004 Superfamily Talitroidea Bulycheva, 1957 Family Talitridae Rafinesque, 1815

#### Floresorchestia

Taxon classificationAnimaliaAmphipodaTalitridae

Bousfield, 1984

##### Type species.

*Orchestia floresiana* Weber 1892, original designation.

##### Species composition.

*Floresorchestia ancheidos* (K.H. Barnard, 1916); *Floresorchestia andrevo* Lowry & Springthorpe, 2015; *Floresorchestia anomala* (Chevreux, 1901); *Floresorchestia anoquesana* (Bousfield, 1971); *Floresorchestia anpingensis* Miyamoto & Morino, 2008; *Floresorchestia australis* Lowry & Springthorpe, 2009b; *Floresorchestia floresiana* (Weber, 1892); *Floresorchestia guadalupensis* Ciavatti, 1989; *Floresorchestia hanoiensis* Hou & Li, 2003; *Floresorchestia kalili* Lowry & Springthorpe, 2015; *Floresorchestia laurenae* Lowry & Springthorpe, 2015; *Floresorchestia malayensis* (Tattersall,1922); *Floresorchestia monospina* (Stephensen, 1935); *Floresorchestia oluanpi* Lowry & Springthorpe, 2015; *Floresorchestia palau* Lowry & Myers, 2013b; *Floresorchestia papeari* Lowry & Springthorpe, 2015; *Floresorchestia pectenispina* (Bousfield, 1970); *Floresorchestia pohnpei* Lowry & Myers, 2013b; *Floresorchestia poorei* Lowry & Springthorpe, 2009a; *Floresorchestia samoana* (Bousfield, 1971); *Floresorchestia samroiyodensis* Azman, Wongkamhaeng & Dumrongrojwattana, 2014; *Floresorchestia seringat* Lowry & Springthorpe, 2015; *Floresorchestia thienemanni* (Schellenberg, 1931); *Floresorchestia vitilevana* (J.L. Barnard, 1960); *Floresorchestia vugiaensis* (Dang & Le, 2011); *Floresorchestia yap* Lowry & Springthorpe, 2015; *Floresorchestia yehyuensis* Miyamoto & Morino, 2008.

##### Key to known Thai Species of *Floresorchestia*

**Table:** 

1	Male gnathopod 1: posterior margin of carpus and propodus each with a lobe covered in palmate seta; uropod 3 peduncle with three robust setae	***Floresorchestia samroiyodensis***
–	Male gnathopod 1 posterior margin of merus, carpus and propodus each with a lobe covered in palmate seta; uropod 3 peduncle with two robust setae	**2**
2	Gnathopod 2 extending between 31–35% along the posterior margin; uropod 1 peduncle with 4-6 robust setae; uropod 3 ramus without marginal robust setae; telson apically incised with four robust setae per lobe	***Floresorchestia boonyanusithii* sp. n.**
–	Gnathopod 2 reaching between 36–40% along posterior margin; uropod 1 peduncle with more than six robust setae; uropod 3 ramus with a marginal robust seta; telson apically incised with five robust setae per lobe	***Floresorchestia buraphana* sp. n.**

#### Floresorchestia boonyanusithii sp. n.

Taxon classificationAnimaliaAmphipodaTalitridae

http://zoobank.org/6DD48CE1-F8DE-41CE-BA9E-3D2A758971FA

**Figure 1.**
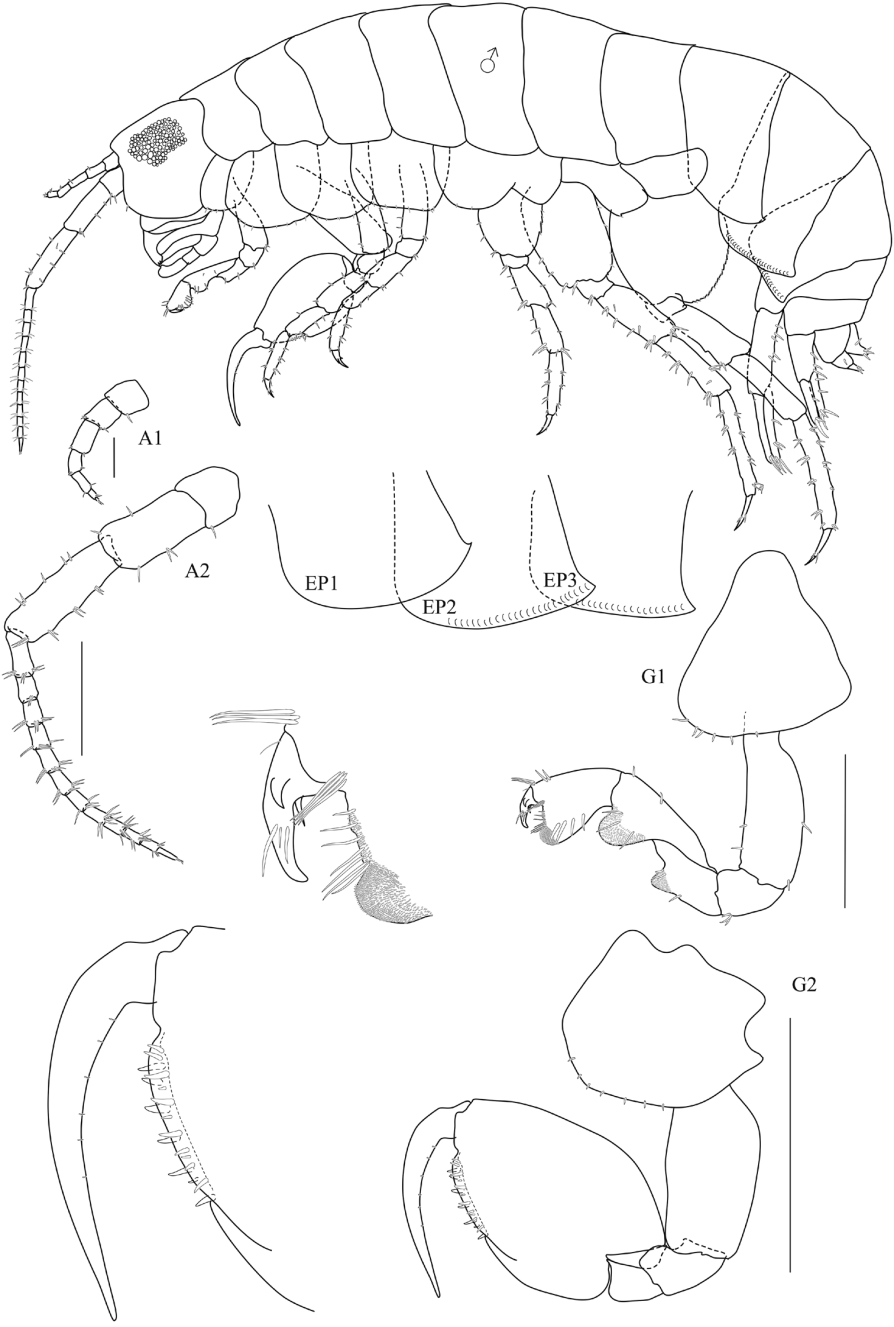
*Floresorchestia boonyanusithii* sp. n. holotype, male, 5.5 mm (PSUZC-CR-0310), Phutsa Reservoir in Nakhon Ratchasima. Scale bars for A2, G1 and G2 represent 0.5 mm and for A1 represents 0.2 mm.

**Figure 2.**
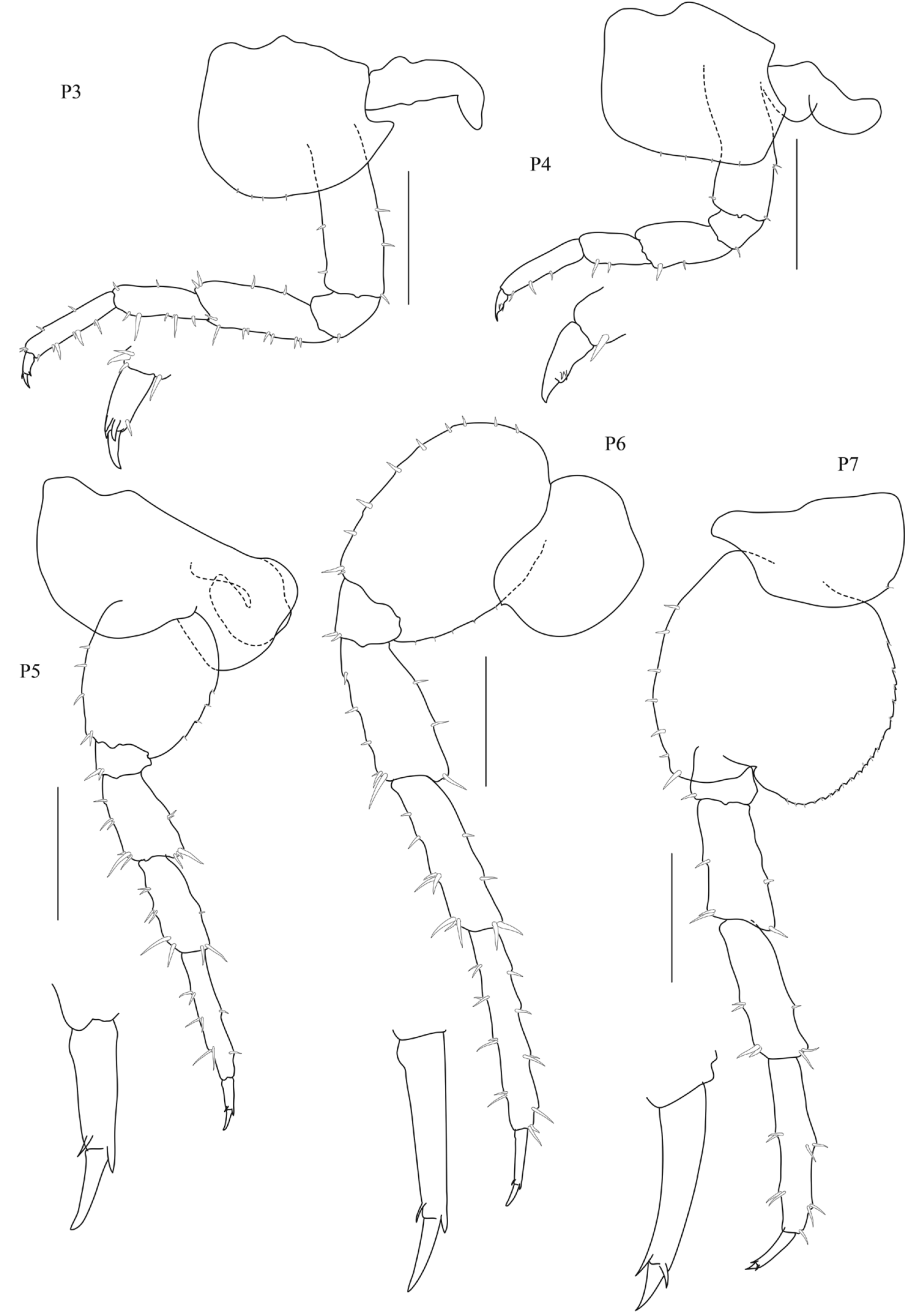
*Floresorchestia boonyanusithii* sp. n. holotype, male, 5.5 mm (PSUZC-CR-0310), Phutsa Reservoir in Nakhon Ratchasima. Scale bars represent 0.5 mm.

**Figure 3.**
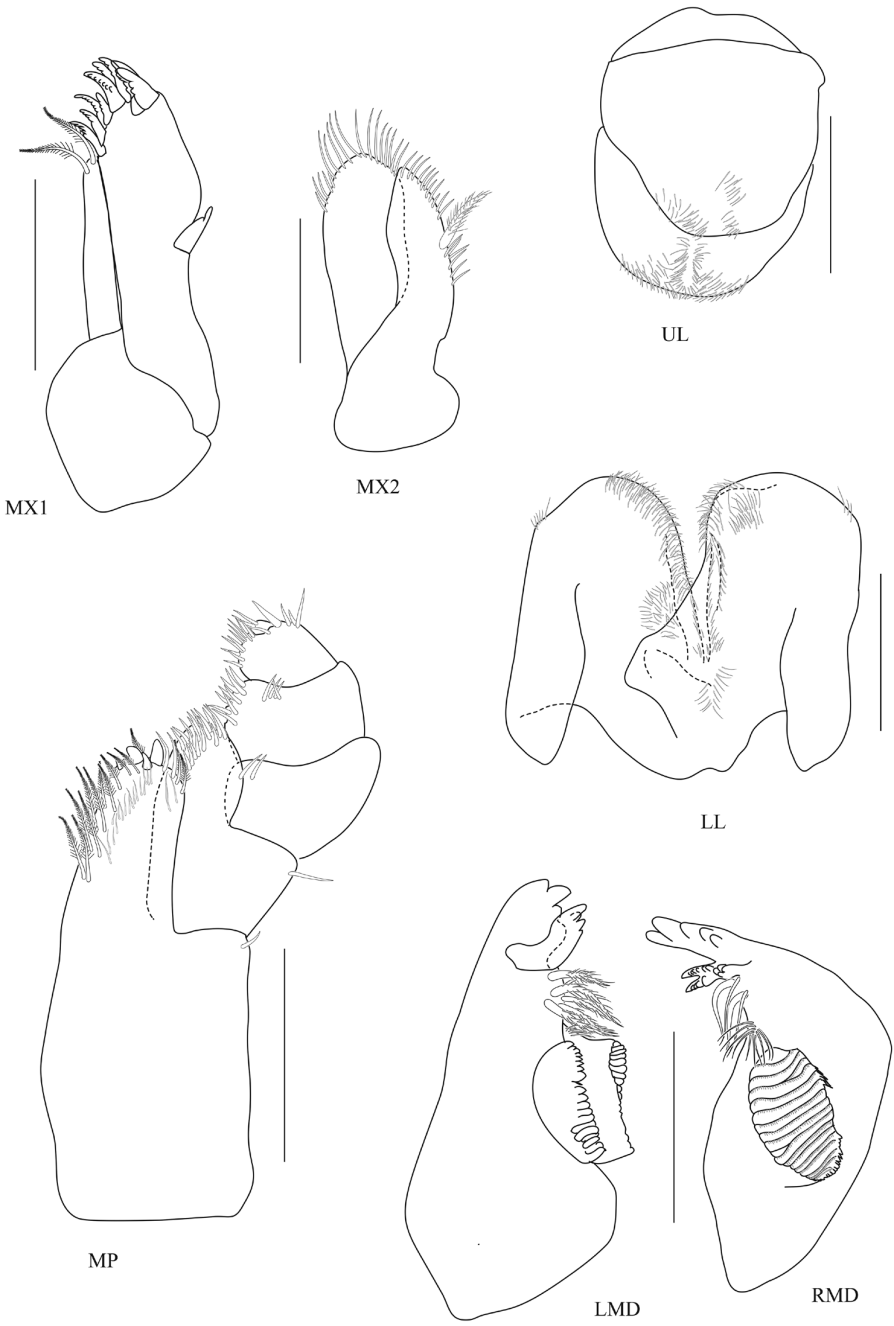
*Floresorchestia boonyanusithii* sp. n. holotype, male, 5.5 mm (PSUZC-CR-0310), Phutsa Reservoir in Nakhon Ratchasima. Scale bars for MX1, MX2, MP, LMD and RMD represent 0.2 mm and for UL and LL represent 0.1 mm.

**Figure 4.**
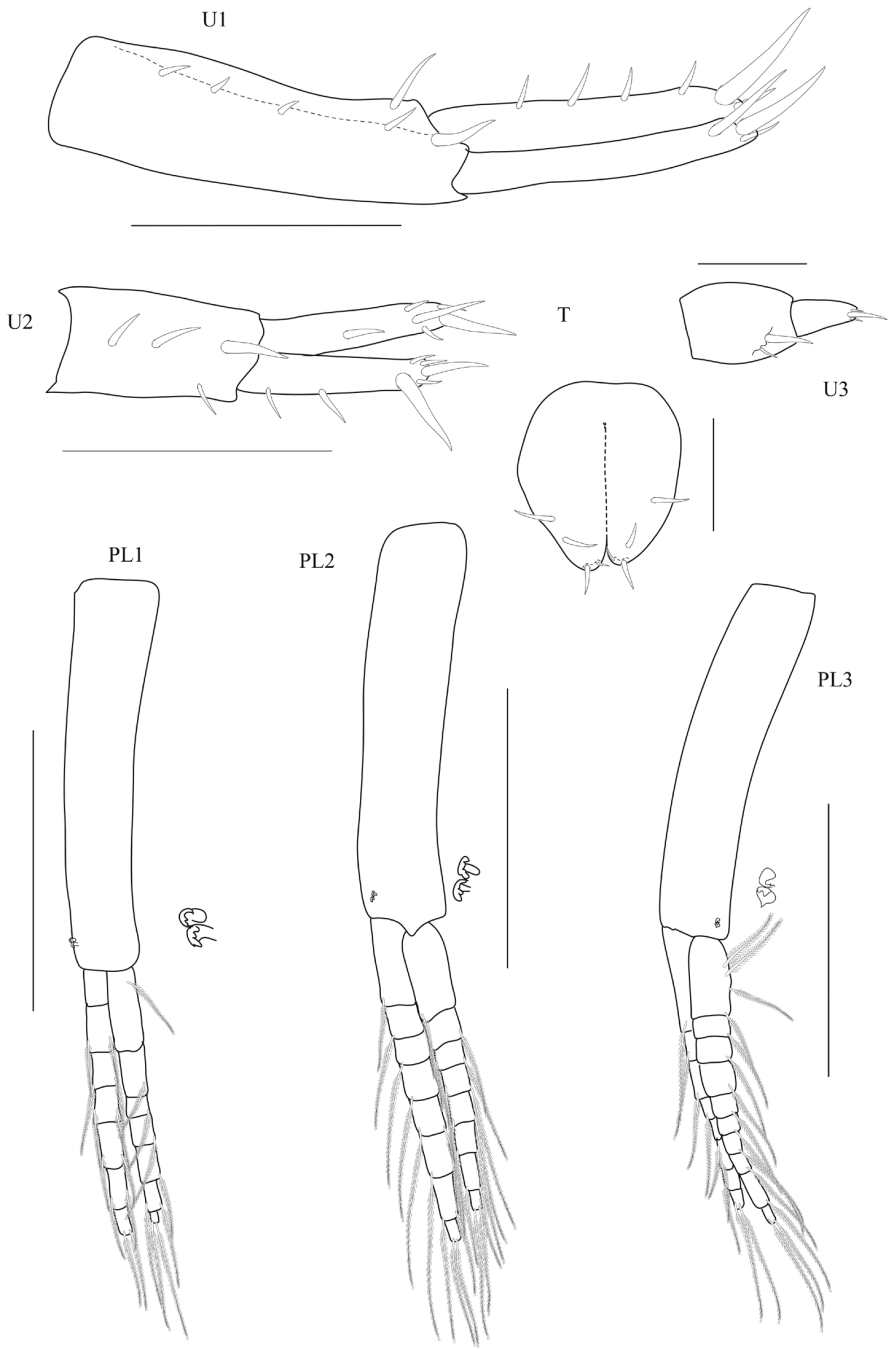
*Floresorchestia boonyanusithii* sp. n. holotype, male, 5.5 mm (PSUZC-CR-0310), Phutsa Reservoir in Nakhon Ratchasima. Scale bars for PL1-3 represent 0.5 mm, for U1-2 represent 0.2 mm, and U3 and T represent 0.1 mm.

**Figure 5.**
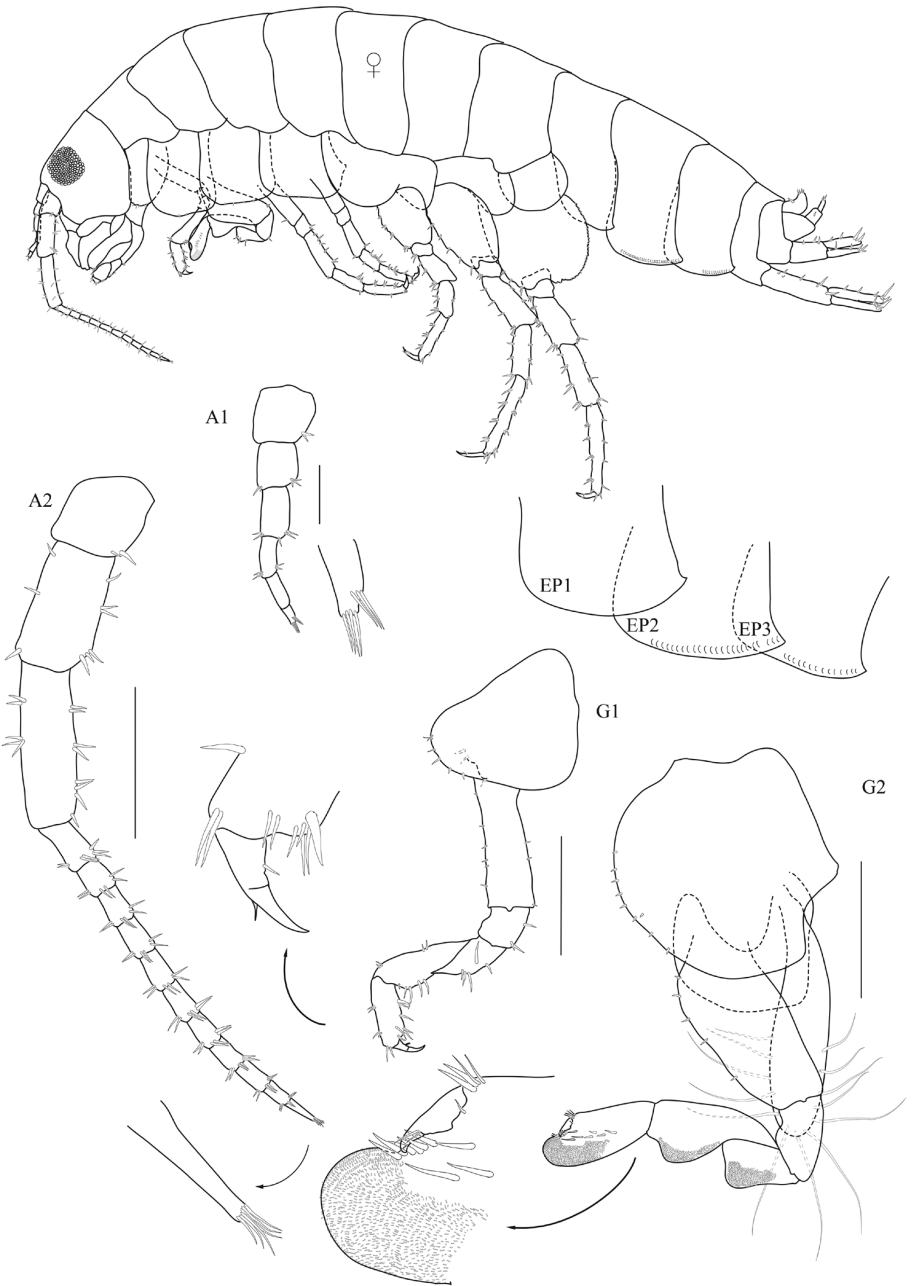
*Floresorchestia boonyanusithii* sp. n. allotype, female 8.8 mm (PSUZC-CR-0311), Phutsa Reservoir in Nakhon Ratchasima. Scale bars for A2, G1 and G2 represent 0.5 mm and for A1 represents 0.2 mm.

Figures 1 , 2 , 3 , 4 , 5 , Table 1

##### Type material.

Holotype. ♂, THAILAND, North-eastern Thailand, Phutsa Reservoir, Nakhon Ratchasima (15°2'59"N, 102°2'18"E), 21 Febuary 2016, Boonyanusith, C. PSUZC-CR-0310. Allotypes, ♀ collected with holotype; PSUZC-CR-0311; Paratype, collected with holotype (PSUZC-CR-0312 (5♂; 5♀))

##### Etymology.

Named after Chaichat Boonyanusith in appreciation of his contribution to the terrestrial amphipod study in Thailand.

##### Ecological type.

Land-hoppers (truly terrestrial). Size 5.5 mm. Sexual dimorphism present.

##### Description.

Based on male, holotype, 5.5 mm, PSUZC-CR 0310.

**Head.** *Eye* large (1/2 head length). *Antenna 1* short, having four short articles of flagellum, extending one quarter (¼) of peduncle article 5. *Antenna 2* peduncular articles slender; article 5 longer than article 4; flagellum of 13 articles, longer than peduncle, final article minute, without apical cluster of serrate setae. *Upper lip* broad, deep, apex rounded. *Lower lip* without inner plates. *Left mandible* incisor 4-dentate, lacinia mobilis 4-dentate, molar strong. *Maxilla 1* inner plate slender with two terminal setae; outer plate with nine terminal serrate setae with small palp, 1-articulate. *Maxilliped* inner plate with apical and subapical plumose setae and three large conical robust setae; outer plate with two rows of subapical setae; palp article 2 distomedial lobe well developed; article 4 reduced, button-shaped.

**Pereon.** *Gnathopod 1* sexually dimorphic; subchelate; coxa 1 smaller than coxa 2, ventral margin with six setae; posterior margin of merus, carpus and propodus each with a lobe covered in palmate setae, palmate lobes in male only; carpus 1.4 times as long as propodus, 2.6 times as long as broad; propodus subtriangular with well-developed posterodistal lobe, anterior margin with two groups of robust setae, posterior margin with four robust setae, palm with seven robust setae; dactylus cuspidactylate, subequal in length to palm.

*Gnathopod 2* sexually dimorphic; subchelate; basis anterior margin smooth, widened distally; ischium with rounded lobe on mid-anterior margin; carpus triangular, reduced (enclosed by merus and propodus) without posterior lobe; propodus subovate, 1.5 times as long as wide; palm acute, extending 33% along posterior margin, lined with robust setae; dactylus longer than palm, attenuated distally, posterior margin smooth.

*Pereopods 3–7* cuspidactylate. *Pereopod 3* coxa subquadrate with posterior process, merus longer than carpus, distally expanded; propodus slender, longer than carpus. *Pereopod 4* slightly shorter than pereopod 3; coxa wider than long with posterior process; carpus significantly shorter than carpus of pereopod 3; dactylus thickened proximally with a notch midway along the posterior margin. *Pereopod 5* coxa bilobed, anterior lobe slightly larger than the posterior lobe; propodus distinctly longer than the carpus. *Pereopods 6–7; pereopod 6* subequal in length to pereopod 7; coxa posterior lobe inner view posteroventral corner rounded, posterior margin perpendicular to ventral margin; coxal gill convoluted; propodus slender, longest; dactylus narrow. *Pereopod 7*; basis lateral sulcus absent, posterior margin with distinct minute serrations, each with a small seta, posterodistal lobe present, shallow, broadly rounded; merus and carpus distally expanded; carpus subrectangular propodus slender, longest; dactylus short.

**Pleon.** *Pleopods* all well developed. *Pleopod 1* peduncle without marginal setae; biramous, inner ramus not longer than outer ramus, shorter than peduncle; inner ramus with seven articles; outer ramus with seven articles. *Pleopod 2* peduncle without marginal setae; biramous, inner ramus subequal to outer, shorter than peduncle; inner ramus with eight articles; outer ramus with eight articles. *Pleopod 3* peduncle without marginal setae; biramous, outer ramus shorter than peduncle; inner ramus with ten articles; outer ramus with eight articles.

*Epimera 1–3* vertical slits present on plate 2 and 3. *Epimera 2* with 27 slits. *Epimera 3* with 20 slits. *Epimeron 2* subequal in length to *epimeron 3*. *Epimeron 3* posterior margin smooth, without setae, posteroventral corner with small subacute tooth, ventral margin without robust setae.

*Uropod 1* peduncle with four robust setae, distolateral robust seta absent; inner ramus subequal in length to outer ramus, inner ramus with marginal robust setae (one row), with four marginal robust setae; outer ramus without marginal robust setae. *Uropod 2* not sexually dimorphic; peduncle with three robust setae; inner ramus subequal in length to outer ramus, with marginal robust setae; outer ramus with marginal robust setae in one row, outer ramus with two marginal robust setae. *Uropod 3* peduncle with two robust setae; ramus not fused to peduncle; ramus shorter than peduncle, 1.75 times as long as broad, ramus linear with three apical setae.

*Telson* longer than broad, apically cleft, dorsal midline half of the telson, with marginal and apical robust setae, four setae per lobe.

**Female (sexually dimorphic characters).** *Gnathopod 1* coxa anterior margin straight; merus lacking tumescent lobe, posterior margin with five robust setae; propodus without tumescent protuberance, anterior margin with two groups of robust setae; palm slightly acute, dactylus inner lateral posterior margin with one robust seta.

*Gnathopod 2* mitten-shaped; posterior margin of carpus and propodus each with lobe covered in palmate setae; carpus well developed, posterior lobe projecting between merus and propodus; nearly twice as long as wide; palm obtuse, not lined with robust setae, posterodistal corner without spine; dactylus subequal in length to palm, not modified distally, blunt.

##### Habitat.

Terrestrial, most prefer living in *Typha angustifolia* root near reservoir.

##### Distribution.

North-eastern Thailand.

##### Remarks.

*Floresorchestia boonyanusithii* sp. n. is the second land-hopper reported in Thailand. It can be distinguished from *Floresorchestia samroiyodensis* by the characters in the above key. Additionally, this species shows the following differences: 1) the left mandible lacinia mobilis has four cusps in both male and female (vs. male 4-dentate and female 6-dentate); 2) male gnathopod 1 carpus is 2.6 times longer than broad, 1.4 as long as propodus (vs. 2 times longer than broad, 1.8 times as long as propodus); 3) male gnathopod 2 propodus 1.4 times longer than broad, palm extending 39% along posterior margin (propodus 1.8 times longer than broad, palm extending 27% along posterior margin); 4) pereopod 7 is shallow, broadly rounded (vs. rounded, produced downwards almost to merus).

*Floresorchestia boonyanusithii* is characterised by a mandible left lacinia mobilis 4-dentate and uropod 3 peduncle with two robust setae. The only similar species are *Floresorchestia buraphana* sp. n. and *Floresorchestia hanoiensis* Hou & Li, 2003. The new species differs from *Floresorchestia hanoiensis* Hou & Li, 2003 in its pereopod 4 is thickened proximally with a notch midway along its posterior margin (vs. pereopod 4 similar to pereopod 3 dactylus) pereopod 7 basis shallow, broadly rounded (vs. produced downwards almost to merus); pleopod 1-3 peduncle without marginal robust setae; epimera 3 posterior margin without setae with 20 slits (vs epimera 3 posterior margin with five slits) and telson apically incised with four robust setae per lobe (vs. three robust setae per lobe).

*Floresorchestia boonyanusithii* shared several characters with *Floresorchestia buraphana* in having a mandible left lacinia mobilis 4-dentate; posterior margin of merus, carpus and propodus covered in palmate setae; uropod 3 peduncle with two robust setae and telson is longer than broad. *Floresorchestia boonyanusithii* differs from *Floresorchestia buraphana* in having uropod 3 rami without marginal setae (vs rami with one marginal seta) and telson with four robust setae per lobe (vs. five robust setae per lobe).

#### Floresorchestia buraphana sp. n.

Taxon classificationAnimaliaAmphipodaTalitridae

http://zoobank.org/ED87B33E-9CB9-4F84-B23D-E325114C9976

**Figure 6.**
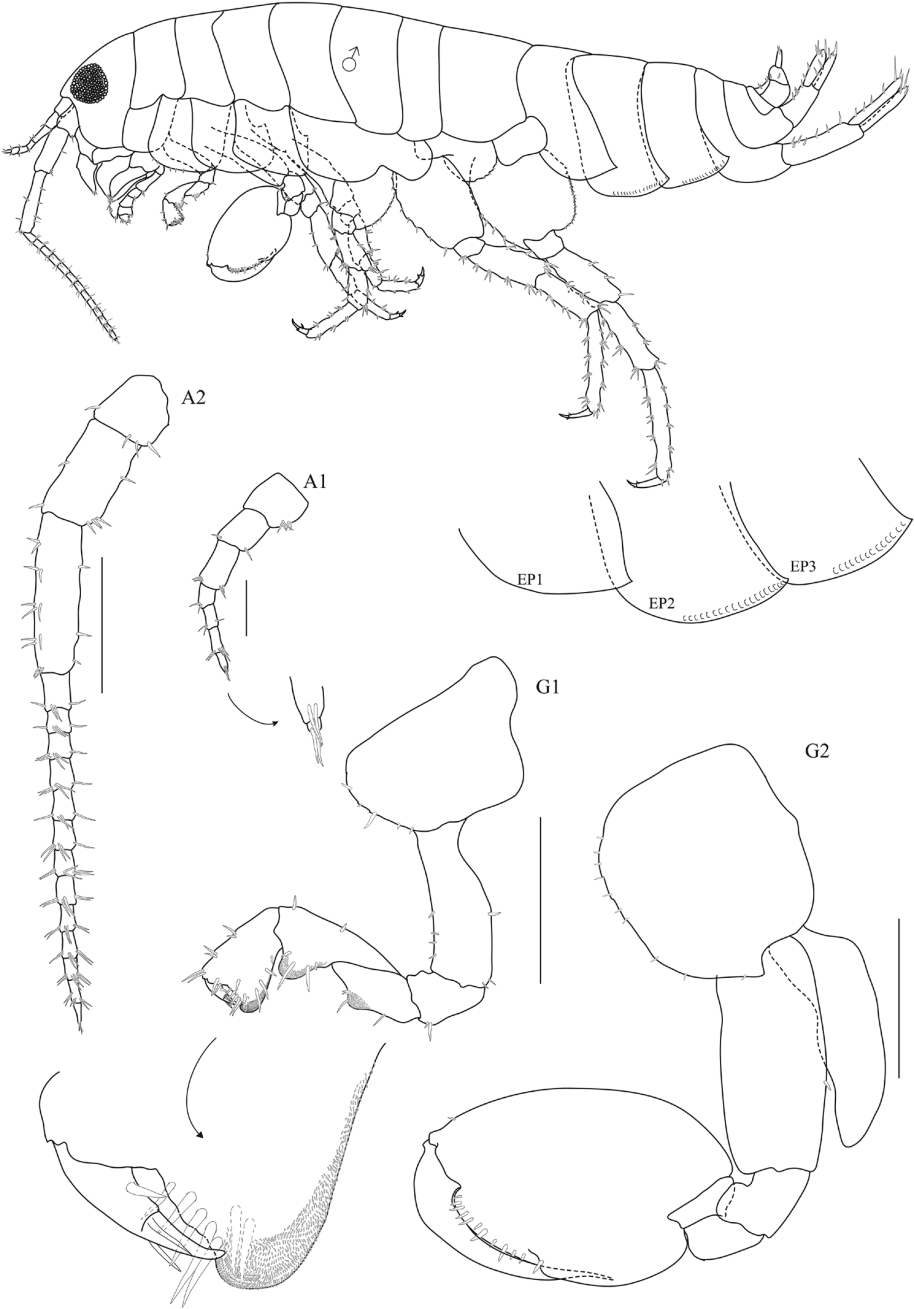
*Floresorchestia buraphana* sp. n. holotype, male, 6.3 mm (PSUZC-CR-0312), Burapha University, Chonburi. Scale bars for A2, G1 and G2 represent 0.5 mm and for A1 represents 0.2 mm.

**Figure 7.**
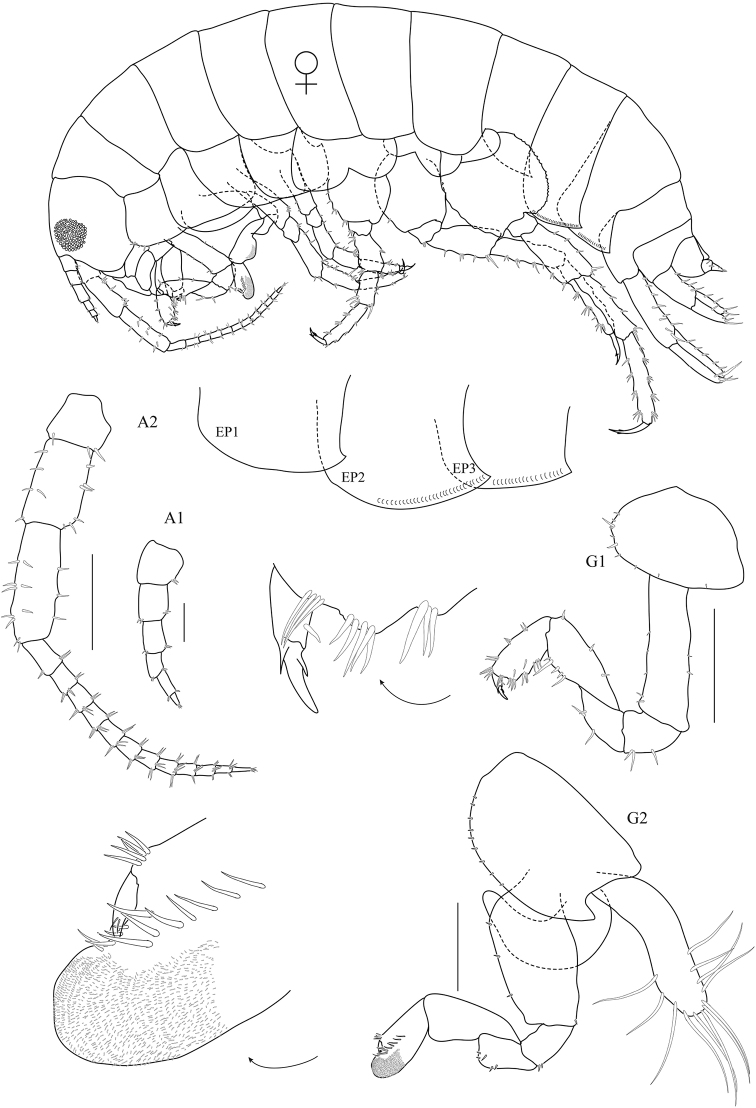
*Floresorchestia buraphana* sp. n. holotype, male, 6.3 mm (PSUZC-CR-0312), Burapha University, Chonburi. Scale bars 0.5 mm.

**Figure 8.**
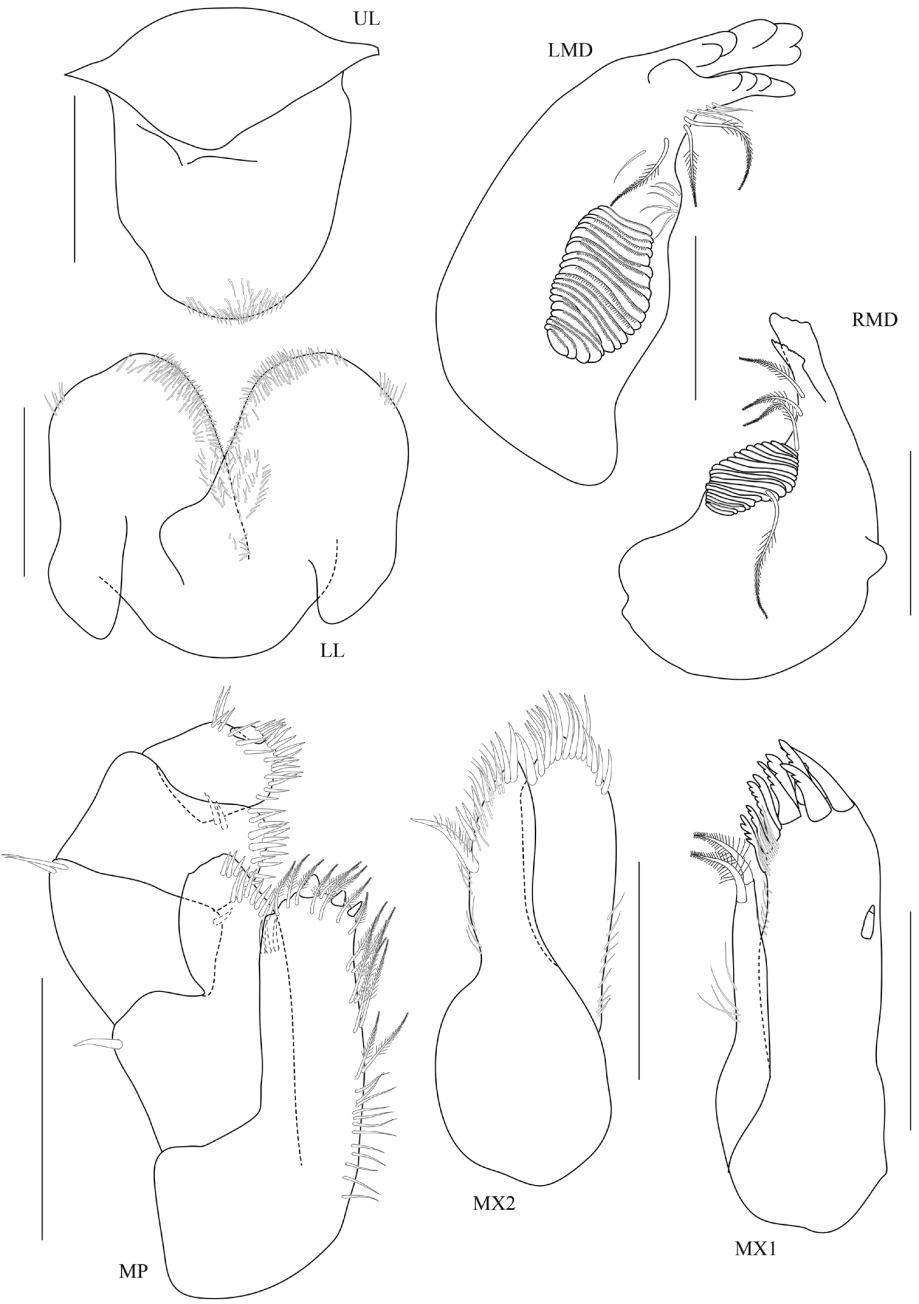
*Floresorchestia buraphana* sp. n. holotype, male, 6.3 mm (PSUZC-CR-0312), Burapha University, Chonburi. Scale bars for MX1, MX2, MP, LMD and RMD represent 0.2 mm and for UL and LL represent 0.1 mm.

**Figure 9.**
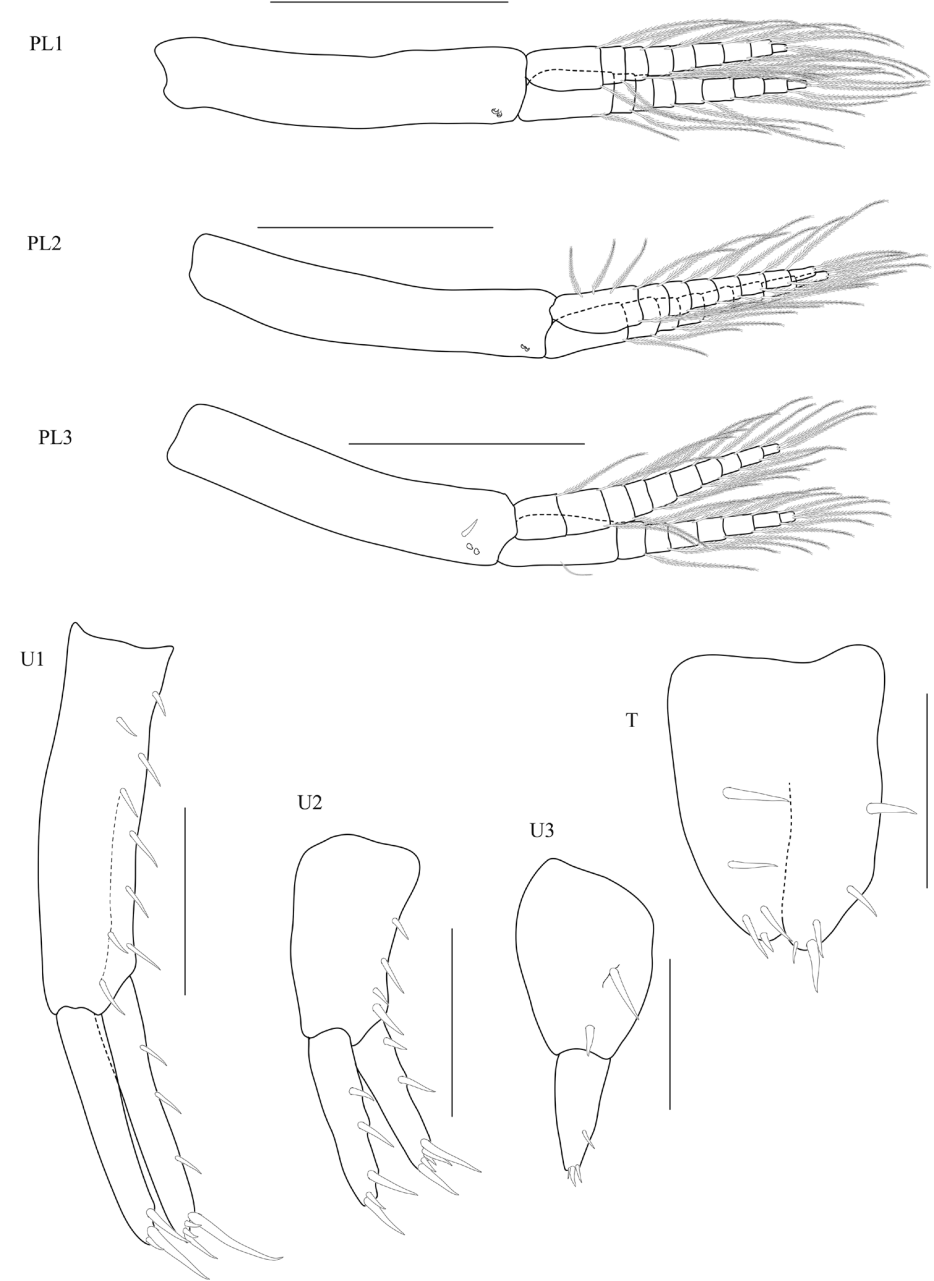
*Floresorchestia buraphana* sp. n. holotype, male, 6.3 mm (PSUZC-CR-0312), Burapha University, Chonburi. Scale bars for PL1-3 represent 0.5 mm, for U1-2 represent 0.2 mm, and for U3 and T represent 0.1 mm.

**Figure 10.**
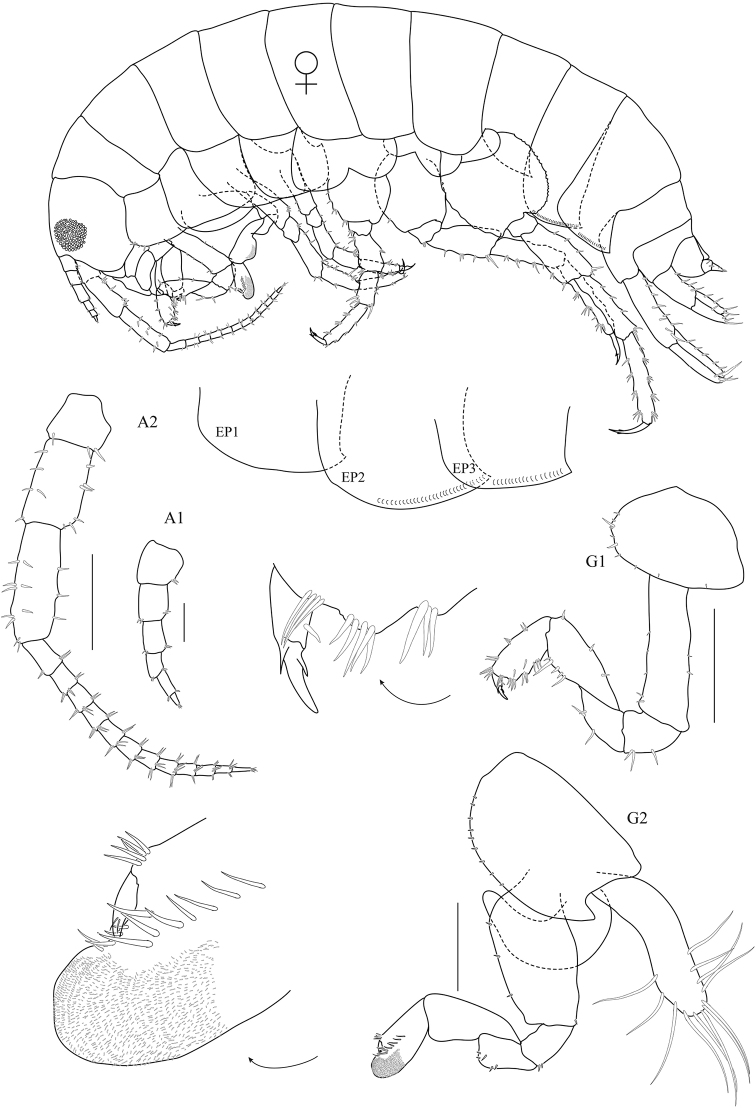
*Floresorchestia buraphana* sp. n. allotype, female 8.8 mm (PSUZC-CR-0313), Burapha University, Chonburi. Scale bars 0.5 mm.

Figures 6 , 7 , 8 , 9 , 10 , Table 1

##### Type material.

Holotype. ♂, THAILAND, Eastern Thailand, Man-made swamp in Burapha University, Chonburi (13°16'37"N, 100°55'21"E), 21 April 2016, Damrongrojwattana, P. PSUZC-CR-0312. Allotypes, ♀ collected with holotype; PSUZC-CR-0313; Paratype, collected with holotype (PSUZC-CR-0314 (5♂; 5♀))

##### Etymology.

Named for Burapha University where the man-made swamp is the type locality of the species.

##### Ecological type.

Supralittoral (325 m from the Bang San Beach). Size 6.3 mm. Sexual dimorphism present.

##### Description.

Based on male, holotype, 6.3 mm, PSUZC-CR 0312.

**Head.** *Eye* large (one third of head length). *Antenna 1* short, having five articles of flagellum, extending one quarter (¼) of antenna 2 peduncle article 5. *Antenna 2* peduncular articles slender; article 5 longer than article 4; flagellum of 13 articles, longer than peduncle, final article minute, without apical cluster of serrate setae. *Upper lip* broad, deep, apex rounded. *Lower lip* without inner plates. *Left mandible* incisor 5-dentate, lacinia mobilis 5-dentate, molar strong. *Maxilla 1* inner plate slender with two terminal setae; outer plate with nine serrate robust setae and with small palp, 1-articulate. *Maxilliped* inner plate with apical and subapical plumose setae and three large conical robust setae; outer plate with two rows of subapical setae; palp article 2 distomedial lobe well developed; article 4 reduced, button-shaped.

**Pereon.** *Gnathopod 1* sexually dimorphic; subchelate; coxa 1 smaller than coxa 2, ventral margin with five setae; posterior margin of merus, carpus and propodus each with lobe covered in palmate setae, palmate lobes in male only; carpus 1.5 times as long as propodus, twice as long as broad; propodus sub-triangular with well-developed posterodistal lobe, anterior margin with two groups of robust setae, posterior margin with five robust setae, palm with five robust setae; dactylus cuspidactylate, subequal in length to palm. *Gnathopod 2* sexually dimorphic; subchelate; basis anterior margin smooth, widened distally; ischium with rounded lobe on mid-anterior margin; carpus triangular, reduced (enclosed by merus and propodus) without posterior lobe; propodus subovate, 1.5 times as long as wide; palm acute, extending 37% along posterior margin, lined with robust setae; dactylus longer than palm, attenuated distally, posterior margin smooth.

*Pereopods 3–7* cuspidactylate. *Pereopod 3* coxa subquadrat with posterior process, merus longer than carpus, distally expanded; propodus slender, longer than carpus. *Pereopod 4* slightly shorter than pereopod 3; coxa wider than long with posterior process; carpus significantly shorter than carpus of pereopod 3; dactylus thickened proximally with a notch midway along posterior margin. *Pereopod 5* coxa bilobed, anterior lobe larger than posterior lobe; propodus distinctly longer than carpus. *Pereopod 6* subequal in length to pereopod 7; coxa posterior lobe inner view - posteroventral corner rounded, posterior margin perpendicular to ventral margin; coxal gill simple; propodus slender, longest; dactylus narrow. *Pereopod 7* basis lateral sulcus absent, posterior margin with distinct minute serrations, each with a small seta, posterodistal lobe present, shallow, broadly rounded; merus and carpus distally expanded; carpus subrectangular, propodus slender, longest; dactylus short.

**Pleon.** *Pleopods* all well developed. *Pleopod 1* peduncle without marginal setae; biramous, inner ramus subequal to outer ramus, shorter than peduncle; inner ramus with nine articles; outer ramus with nine articles. *Pleopod 2* peduncle without marginal setae; biramous, inner ramus subequal to outer, shorter than peduncle; inner ramus with eight articles; outer ramus with eight articles. *Pleopod 3* peduncle without marginal setae; biramous, outer ramus shorter than peduncle; inner ramus with eight articles; outer ramus with eleven articles.

*Epimera 1–3* vertical slits present on plates 2 and 3. *Epimera 2* with 27 slits. *Epimera 3* with 20 slits. *Epimeron 2* subequal in length to *epimeron 3*. *Epimeron 3* posterior margin smooth, without setae, posteroventral corner with small subacute tooth, ventral margin without robust setae.

*Uropod 1* peduncle with nine robust setae, distolateral robust seta absent; inner ramus subequal in length to outer ramus, inner ramus with marginal robust setae (one row), with three marginal robust setae; outer ramus without marginal robust setae. *Uropod 2* not sexually dimorphic; peduncle with four robust setae; inner ramus subequal in length to outer ramus, with marginal robust setae; outer ramus with marginal robust setae in one row, outer ramus with two marginal robust setae. *Uropod 3* peduncle with two robust setae; ramus not fused to peduncle; ramus shorter than peduncle, 2.2 times as long as broad, ramus linear with a marginal seta and three apical setae.

*Telson* longer than broad, apically cleft, dorsal midline half of the telson, with marginal and apical robust setae, five setae per lobe.

**Female (sexually dimorphic characters).** *Gnathopod 1* coxa anterior margin straight; merus lacking tumescent lobe, posterior margin with five robust setae; propodus without tumescent protuberance, anterior margin with two groups of robust setae; palm slightly acute, dactylus inner lateral posterior margin with one robust seta.

*Gnathopod 2* mitten-shaped; posterior margin of carpus and propodus each with a lobe covered in palmate setae; carpus well developed, posterior lobe projecting between merus and propodus; nearly twice as long as its width; palm obtuse, not lined with robust setae, posterodistal corner without spine; dactylus subequal in length to palm, not modified distally, blunt.

##### Habitat.

Fresh water swamps in Burapha University.

##### Distribution.

Eastern Thailand.

##### Remarks.

The characters of *Floresorchestia buraphana* sp. n. that separate it from *Floresorchestia samroiyodensis* Azman et al. 2014 and *Floresorchestia boonyanusithii* sp. n. have been given in the above key. Additionally, this species shows the following features: 1) uropod 1 peduncle with nine robust setae; 2) uropod 3 peduncle with two robust setae and rami with three apical setae and telson longer than broad, dorsal midline at least halfway, apically incised with five robust setae per lobe.

*Floresorchestia buraphana* sp. n. appears to be closely related to *Floresorchestia yehyuensis*, sand-hopper from Taiwan in having; 1) a mandible left lacinia mobilis 4-dentate; 2) gnathopod 1 merus, carpus and propodus each with palmate lobe and 3) uropod 3 ramus with one marginal setae. It differs however in the pereopod 4 dactylus which is thickened proximally with a notch midway along the posterior margin (similar to pereopod 3 dactylus in *Floresorchestia yehyensis*), uropod 3 peduncle with three robust setae (with two robust setae in *Floresorchestia yehyensis*), epimera 2 with 25 slits (33 slits in *Floresorchestia yehyensis*) and telson longer than broad with dorsal midline half way (telson is broader than its length without dorsal midline in *Floresorchestia yehyensis*).

**Table 1.** A summary of the diagnostic characteristics that serve to distinguish closely related *Floresorchestia* species.

Species	Body length (mm)	Left mandible lacinia mobilis	Gnathopod 1	Gnathopod 1 carpus	Pleopod 1–3 peduncle	Slit on epimera 2 and 3	Uropod 1 peduncle	Uropod 3 peduncle	Telson
*Floresorchestia boonyanusithii* sp. n.	5.5	4-dentate	Posterior margin of merus, carpus and propodus each with lobe covered in palmate setae	2.6 × as long as broad, palm extending 33% along posterior margin	Without marginal setae	27 and 20 slits	With 6 robust setae	Peduncle with 2 robust setae, rami 1.8 as long as broad without marginal setae	Dorsal midline at least halfway with 4 setae per lobe
*Floresorchestia buraphana* sp. n.	6.3	4-dentate	Posterior margin of merus, carpus and propodus each with lobe covered in palmate setae	2 × as long as broad, palm extending 37% along posterior margin	Without marginal setae	25 and 15 slits	With 9 robust setae	Peduncle with 2 robust setae, rami 2.2 x as long as broad with a marginal seta	Dorsal midline at least halfway with 5 setae per lobe
*Floresorchestia hanoiensis* Hou & Li, 2003	6.5	4-dentate	Posterior margin of merus, carpus and propodus each with lobe covered in palmate setae	1.9 × as long as broad, palm extending between 46-50% along posterior margin	With marginal robust setae	22 and 5 slits	With 8 robust setae	Peduncle with 2 robust setae, rami 1.8 as long as broad without marginal setae	Dorsal midline entire with 3 setae per lobe
*Floresorchestia samroiyodensis* Azman, Wongkamhaeng & Dumrongrojwattana, 2014	10.5	Male 4-dentate, female 6-dentate	Posterior margin of carpus and propodus each with lobe covered in palmate setae	2 × as long as broad, palm extending 27% along posterior margin	Without marginal setae	21 and 13 slits	With 6 robust setae	Peduncle with 2 robust setae, rami 1.9 as long as broad with a marginal seta	Dorsal midline vestigial or absent with 5 setae per lobe

## Supplementary Material

XML Treatment for Floresorchestia

XML Treatment for Floresorchestia boonyanusithii

XML Treatment for Floresorchestia buraphana
